# Supplementary material for: Evaluating the Transition from Targeted to Exome Sequencing: A Guide for Clinical Laboratories
Source: Int J Mol Sci. 2023 Apr 15;24(8):7330. doi: 10.3390/ijms24087330 (PMC10138641; doi:10.3390/ijms24087330)
Supplement: Supplementary file 1 [file ijms-24-07330-s001.zip › ijms-2316103-SI.pptx]

## Slide 1
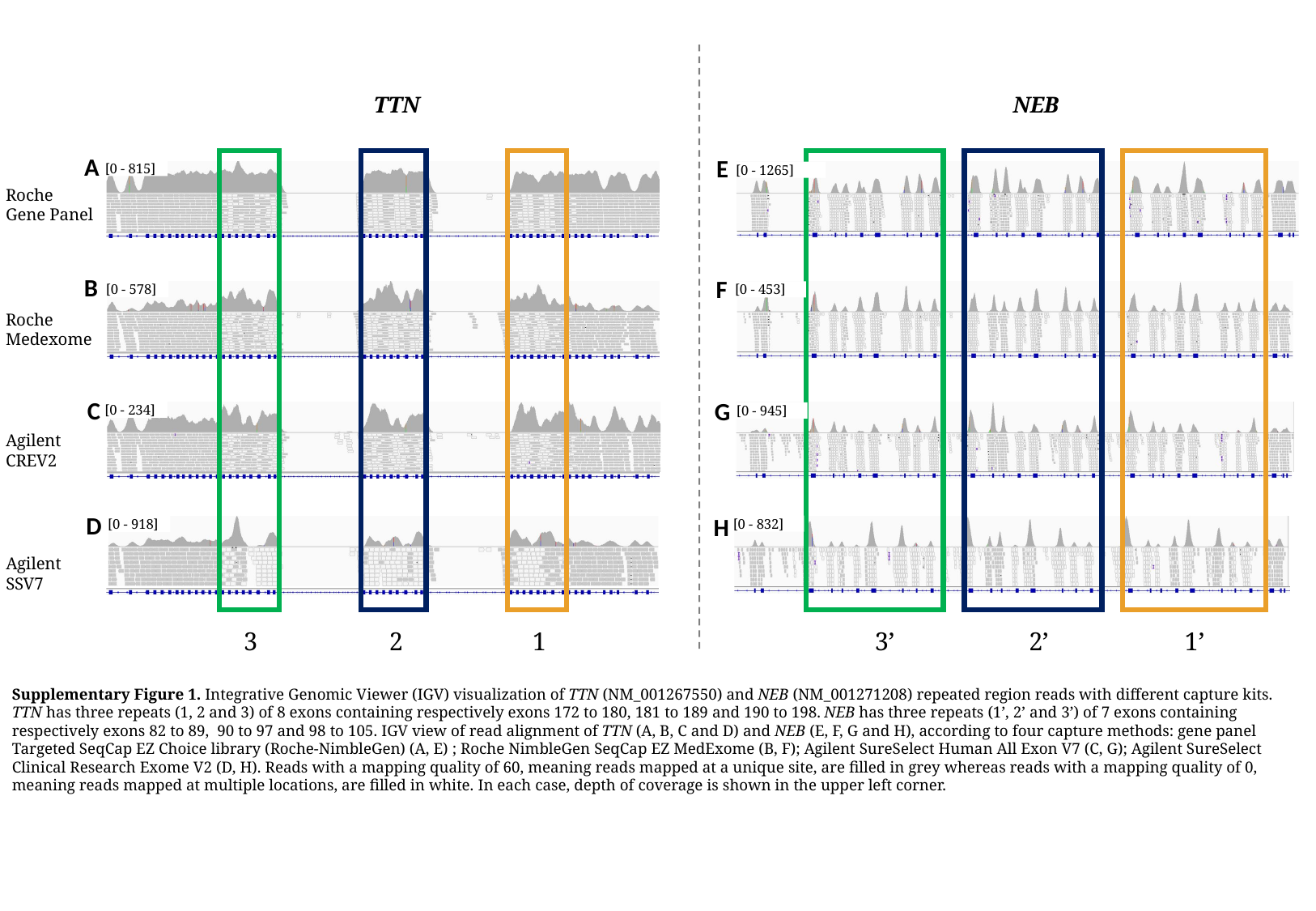

TTN
NEB
A
E
[0 - 815]
[0 - 1265]
Roche
Gene Panel
B
F
[0 - 578]
[0 - 453]
Roche
Medexome
C
G
[0 - 234]
[0 - 945]
Agilent
CREV2
D
H
[0 - 832]
[0 - 918]
Agilent
SSV7
1’
1
3’
3
2
2’
Supplementary Figure 1. Integrative Genomic Viewer (IGV) visualization of TTN (NM_001267550) and NEB (NM_001271208) repeated region reads with different capture kits. TTN has three repeats (1, 2 and 3) of 8 exons containing respectively exons 172 to 180, 181 to 189 and 190 to 198. NEB has three repeats (1’, 2’ and 3’) of 7 exons containing respectively exons 82 to 89, 90 to 97 and 98 to 105. IGV view of read alignment of TTN (A, B, C and D) and NEB (E, F, G and H), according to four capture methods: gene panel Targeted SeqCap EZ Choice library (Roche-NimbleGen) (A, E) ; Roche NimbleGen SeqCap EZ MedExome (B, F); Agilent SureSelect Human All Exon V7 (C, G); Agilent SureSelect Clinical Research Exome V2 (D, H). Reads with a mapping quality of 60, meaning reads mapped at a unique site, are filled in grey whereas reads with a mapping quality of 0, meaning reads mapped at multiple locations, are filled in white. In each case, depth of coverage is shown in the upper left corner.
